# ASSOCIATIONS OF PROTEIN INTAKE WITH THE RISK OF ALL-CAUSE, CARDIOVASCULAR, AND CANCER MORTALITY: A META-ANALYSIS

**DOI:** 10.1093/geroni/igae098.3007

**Published:** 2024-12-31

**Authors:** Huili Yang, Meng Cao, Chongmei Huang, Yaping Jin, Minhui Liu

**Affiliations:** Ningxia Medical University, Yinchuan, Ningxia, China (People’s Republic); Ningxia Medical University, Yinchuan, Ningxia, China; Ningxia Medical University, Yinchuan, Ningxia, China (People’s Republic); Ningxia Medical University, Yinchuan, Ningxia, China; Ningxia Medical University, Yinchuan, Ningxia, China

## Abstract

The association of the amount and sources of protein consumption with mortality for cardiovascular diseases (CVD) and cancer has been extensively studied. However, the outcomes remains controversial. The present meta-analysis was conducted to synthesize findings on the associations between total, animal, and plant protein intake and the risk of mortality from all causes, and cancer. Pubmed and Web of Science were searched to identify prospective cohort studies published up to November 2022. Random effects models were used to calculate pooled effect sizes and 95% confidence intervals for the highest versus lowest categories of protein intake. Heterogeneity were evaluated with I2 statistic, and publication bias was evaluated with funnel plots and Egger’s test. A total of 28 cohort studies were identified. The results showed that total protein (HR=0.99, 95%CI: 0.93, 1.04; HR=0.95, 95%CI: 0.87, 1.02; HR=0.97, 95%CI: 0.84, 1.11), animal protein (HR=1.02, 95% CI: 0.98, 1.07; HR=1.05, 95% CI: 0.99, 1.12; HR=1.00, 95% CI: 0.98, 1.02) were not associated with the all-cause, CVD and cancer mortality. However, the intake of plant protein was inversely associated with the risk of all-cause (HR=0.92, 95% CI: 0.87, 0.96) and CVD mortality (HR=0.87, 95% CI:0.81, 0.93), but not cancer mortality (HR=0.98, 95% CI: 0.94, 1.01). This study demonstrates that higher plant protein intake is associated with a reduced risk of all-cause and CVD mortality. Increasing plant protein intake is a potential way to decrease the risk of death.

